# Effects of a Non Thermal Plasma Treatment Alone or in Combination with Gemcitabine in a MIA PaCa2-luc Orthotopic Pancreatic Carcinoma Model

**DOI:** 10.1371/journal.pone.0052653

**Published:** 2012-12-26

**Authors:** Laura Brullé, Marc Vandamme, Delphine Riès, Eric Martel, Eric Robert, Stéphanie Lerondel, Valérie Trichet, Serge Richard, Jean-Michel Pouvesle, Alain Le Pape

**Affiliations:** 1 Centre d’Imagerie du Petit Animal (CIPA) TAAM, UPS44 CNRS, Orléans, France; 2 Centre de Recherche Biologique (CERB), Baugy, France; 3 GREMI UMR-7344 CNRS, Université d'Orléans, Orléans, France; 4 Centre d’Etude des Pathologies Respiratoires (CEPR), INSERM UMR1100-EA6305, Université François Rabelais, Tours, France; 5 INSERM, UMR957, Université de Nantes, Faculté de médecine, Nantes, France; 6 GERMITEC SAS, Clichy, France; B.C. Cancer Agency, Canada

## Abstract

Pancreatic tumors are the gastrointestinal cancer with the worst prognosis in humans and with a survival rate of 5% at 5 years. Nowadays, no chemotherapy has demonstrated efficacy in terms of survival for this cancer. Previous study focused on the development of a new therapy by non thermal plasma showed significant effects on tumor growth for colorectal carcinoma and glioblastoma. To allow targeted treatment, a fibered plasma (Plasma Gun) was developed and its evaluation was performed on an orthotopic mouse model of human pancreatic carcinoma using a MIA PaCa2-luc bioluminescent cell line. The aim of this study was to characterize this pancreatic carcinoma model and to determine the effects of Plasma Gun alone or in combination with gemcitabine. During a 36 days period, quantitative BLI could be used to follow the tumor progression and we demonstrated that plasma gun induced an inhibition of MIA PaCa2-luc cells proliferation *in vitro* and *in vivo* and that this effect could be improved by association with gemcitabine possibly thanks to its radiosensitizing properties.

## Introduction

Developments in plasma physics make possible to use Non Thermal Plasma (NTP) in cancer research. Indeed, the first demonstration of plasma antitumor activity has been brought on subcutaneous tumors submitted to exposure *via* a large surface probe [1], the plasma being delivered in close vicinity of the powered electrode. Recently, improvements in fibered plasma generation allowed a propagation inside long, flexible and small capillaries using gas flow as low as few sccm. This new NTP source, so called Plasma Gun (PG) [2], is one of the large variety of plasma jets exhibiting unique features for biomedical applications, mainly endoscopic administration and in situ treatment of a variety of tumors.

Pancreatic tumors are one of the most lethal types of cancer. Treatments depend on the diagnosis stage, and are mainly based on a tumor resection followed by ionizing radiation and chemotherapy such as gemcitabine (2′2′-difluorodeoxycytidine). Despite these therapeutic approaches, only about 5% of the patients are still alive 5 years after being diagnosed. In this context, localized treatments such as photodynamic therapy are currently under investigations. Photodynamic therapy uses light emission at a specific wavelength combined with a photosensitizing agent in order to generate Reactive Oxygen Species (ROS) in the treated area. For this purpose, specific agents are needed to target the tumor and to avoid systemic side effects such as those associated to sun exposure of the patient. Based on the exploitation of ROS properties, Plasma gun is a potential alternative for a loco regional treatment with *a priori* limited side effects. NTP is an ionized gas (air or noble gas) sustained by a pulsed electric discharge. It is known to have an antitumor effect *in vitro* on various cell lines and *in vivo* on heterotopic xenograft tumors [3]. This cold excited gas (<40°C) contains free charges (electrons, ions), free radicals, excited molecules and can be delivered through capillaries to induce ROS generation in a localized site. Indeed, in a previous study, an increase in mice survival was observed with NTP on U87 human malignant glioma and melanoma heterotopic xenografts [3]–[5]. This antitumor effect was demonstrated to be associated to ROS generation in the vicinity of the cells, leading to DNA damages, cells cycle arrest and finally apoptosis induction with limited side effects to healthy tissues [4], [6].

In a context of translational research and considering the ability of plasma gun to deliver *in situ* exposure to a tumor, a bioluminescent orthotopic pancreatic tumor was developed to allow a longitudinal non-invasive follow up of tumor growth in a clinically relevant animal model. In human, the most common pancreatic cancer is ductal adenocarcinoma [7], developing within the exocrine part of the pancreas and characterized by a severe tumor hypoxia [8] inducing a resistance against chemo- and radiation therapies [9]. In this goal, among other pancreatic tumor cell lines, the MIA PaCa2 cell line appeared quite relevant for a preclinical approach. The effects of PG applied directly on primary pancreatic tumors were compared to gemcitabine as a reference drug [10]. Moreover, considering similarities between plasma and radiotherapy mechanisms associated to the radiosensitizing properties of gemcitabine [11], the potential interest of an association with plasma was investigated.

First we developed a bioluminescent orthotopic model of pancreatic cancer by modifying pancreatic adenocarcinoma cells (MIA PaCa2) to express luciferase gene and then we evaluated limits of this model. Once the model was well characterized, we documented the NTP and gemcitabine antitumor efficacy *in vitro* on MIA PaCa2 cells, and after we evaluated these treatments *in vivo.* So, a luciferase stable transduction of MIA PaCa2 cells was done to allow tumor growth monitoring by *in vivo* bioluminescence imaging (BLI). Interests and limitations of BLI, an imaging modality dependent upon cells metabolism and proliferation, were considered more especially in the context of this hypoxic tumor.

## Materials and Methods

### Drugs and Chemicals

Gemcitabine was purchased from Sigma (Sigma-Aldrich, Lyon, France) and dissolved in sterile NaCl 0,9%. Dulbecco Modified Eagle Medium (DMEM), fetal bovine serum, horse serum, L-glutamine (2 mM), penicillin (50 IU/ml), and streptomycin (50 µg/ml) were from VWR (VWR International S.A.S, France). All other chemicals were from Sigma.

### Culture of MIA PaCa2 Cells

The MIA PaCa2 pancreatic cancer cell line [12] was obtained from the American Type Culture Collection (Rockville, MD). Cells were maintained in a humidified incubator at 37°C in 5% CO_2_ with Dulbecco Modified Eagle medium supplemented with 10% heat-inactivated fetal bovine serum, 2,5% horse serum, 1% L-Glutamine and 1% penicillin and streptomycin.

### Generation of Luciferase Expressing MIA PaCa-2 Cells

To allow bioluminescence imaging (BLI), the MIA PaCa2 cell line was stably transducted with firefly luciferase encoding lentiviral particles as previously described [13]. Briefly, HEK293FT cells (Human Embryonic Kidney cells optimized for viral production) were transfected with 3 µg of the optimized packaging mix from ViraPower Lentiviral Expression System (Invitrogen Life Technologies, Cergy-pontoise, France), 9 µg of pLNT-LucF [13], [14] and 0,9 µg of the pFG12 [15] which encode the luciferase and the Enhanced Green Fluorescent Protein (EGFP), respectively. Virus-containing supernatants were harvested 48 hr post-transfection and concentrated 60-fold by ultrafiltration. To generate stably modified pancreatic adenocarcinoma cells, 10 000 MIA PaCa2 cells were seeded in wells of a 96-well plate in 100 µl medium and infected 24 hr later with 50 µl virus containing supernatant, corresponding to 250 EGFP viral units per cell. After 2 weeks, EGFP expression level was quantified by flow cytometry. In addition, luciferase activity was measured for varying numbers of cells in a 96-well optical bottom plate (NUNC, Dominique Dutscher, Issy les Moulineaux, France) with 50 µl of lysis substrate buffer from the ‘Steady-Glo Luciferase Assay System’ (Promega) in each well. Light measurements were carried out in duplicate by spectrophotometry (VICTOR plate reader, Perkin Elmer, Woodbridge, ON, Canada) for 10 s and results were corrected for background luminescence from parental cells.

### Plasma Gun Source

The Plasma Gun (PG) is a NTP source allowing for the propagation of rare gas plasmas (helium, neon, argon) inside long and thin dielectric capillaries, likely to be used in endoscopic protocols, and the transfer of these plasmas in ambient air at the outlet of the dielectric guide. The rare gas plasma is thus enriched with air species leading to the generation of so called ROS and RNS acting on the target, i.e. Petri dish surface or mouse tissue in this work. Besides ROS and RNS, rare gas excited species, charge particles, UV photons and transient electric fields associated with the propagation of the plasma towards the target are also delivered on the sample. While being one among the large variety of plasma jets developed for a few years in relation with the emerging plasma medicine field, the PG offers unique features for *in vivo* applications. Basically, the plasma is generated in a Dielectric Barrier Discharge (DBD) reactor, consisting in a glass capillary equipped with both an inner hollowed powered electrode and an outer grounded electrode, operating at a voltage of 13 kV and a frequency of 2 kHz. This device is flushed with very small helium flow rates (480 sccm) in comparison with most of other plasma jets operated with flow rate of a few liters per minute. The tailoring of the high voltage pulses applied across the DBD electrodes allows for the generation and propagation at large distances, up to a few meters, of the plasma. In such a way, the plasma delivery occurs at large distances from the high voltage source and consists in truly ambient temperature plasma with no stringent requirement for distance to target or “cooling” gas flow rate matching.

### Animals and Orthotopic Tumor Induction

Female Swiss *nude* mice (Charles River Laboratoires France - L’Arbresle, France) were acclimated for 5 days in the laboratory before experimentation.

Animals were housed in plastic cages inside a controlled ventilated rack with free access to water and food *ad libidum*. All experiments were performed in accordance with national animal care guidelines (EC directive 86/609/CEE, French decree no 87-848), and were approved by the committee on the ethics of Animal Experiments of the CERB (Baugy, France). Tumor xenografts, plasma treatment and BLI were carried out under general anesthesia obtained with 2,5% isoflurane in air (Aerrane®, Maurepas, France) and all efforts were made to minimize suffering.

For tumor orthotopic xenografts, abdomens were prepped with betadine solution. A 1-cm wide incision was made in the left upper quadrant of the abdomen. The tip of pancreatic tail was gently grasped and pancreas/spleen were externalized in a lateral direction to be fully exposed. To improve injection reproducibility, a micromanipulator and a stereomicroscope, were used, the needle being inserted into the tail of pancreas and positioned in the pancreatic head region. 2.10^6^ MIA PaCa2-luc cells in 50 µL PBS were slowly injected using a 27-gauge needle. The spleen was then returned to the appropriate position in abdomen, and skin and peritoneum closed with 5-0 vicryl sutures. The animals were then placed on a warming blanket until they recovered from anesthesia.

All along the study, body weight was followed as an indicator of the health status.

### Gemcitabine and NTP Treatment

For *in vitro* assays, MIA PaCa2 cells (5×10^4^) were seeded in 24- or 96-well plates 24 hr before gemcitabine or NTP treatment that was performed in open air, 2 mm above the surface of the medium (500 µl) containing adherent cells. Gemcitabine was dissolved directly in the medium at different doses (1–200 nM).

Concerning *in vivo* studies, four days after surgical implantation of MIA PaCa2-luc cells, mice were randomized according to bioluminescence and assigned into 4 groups of 8 mice each. The first group was negative control (CTRL) and did not receive treatment but vehicle (saline) and helium gas flow alone required for plasma. Mice in the second group (GEM) received every 5 days gemcitabine *via* the dorsal tail vein at a dose of 200 mg/kg. Dosing was performed during 20 days (5 treatments).

**Figure 1:**
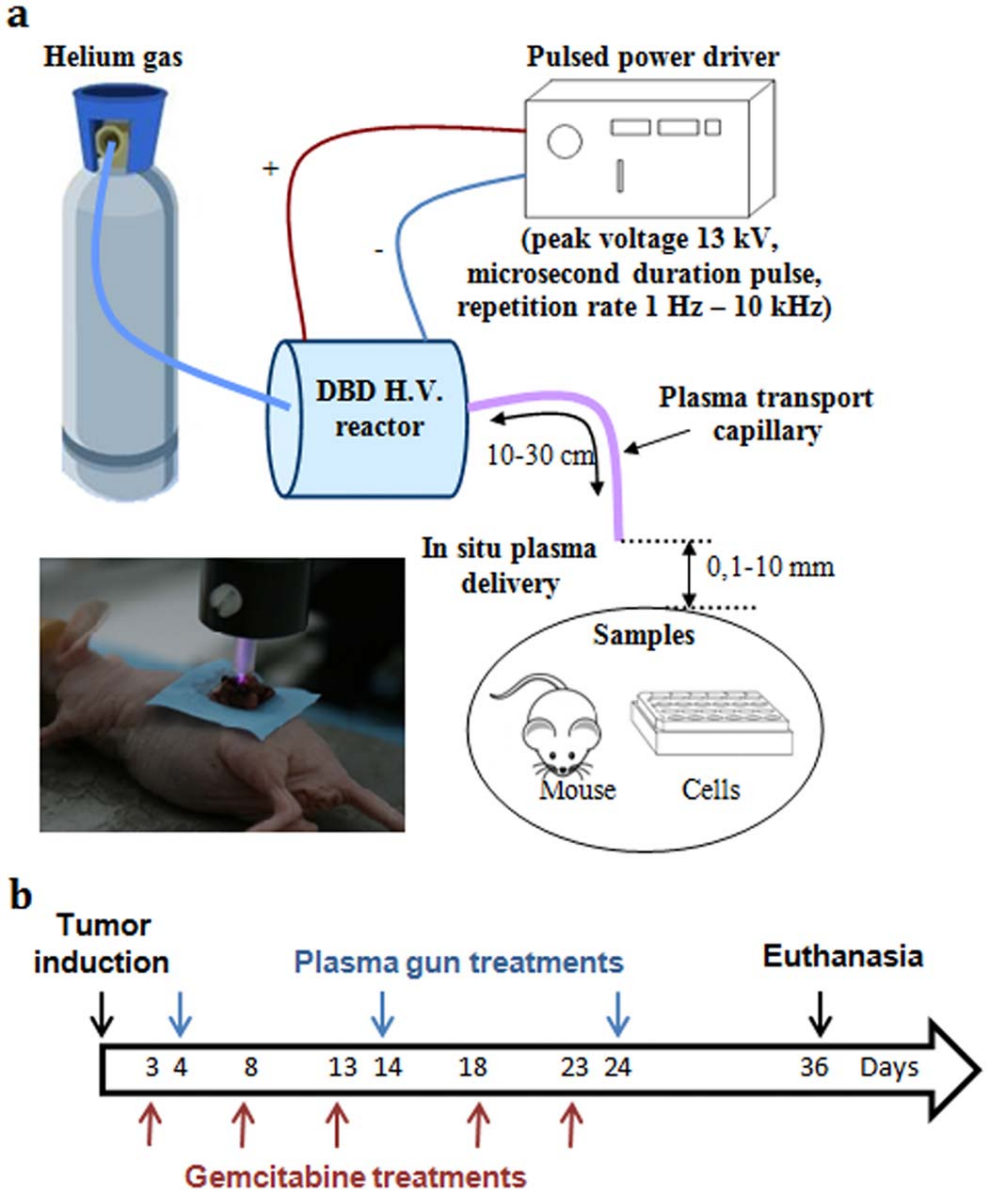
a) Schematics of the plasma gun setup; b) Therapeutic schedule of gemcitabine and plasma gun.

In the third group (NTP) mice received PG three times, at a repetition rate of 2 kHz during 10 min every 10 days, treatment time and frequency used for PG (10 min, 2 kHz) were chosen based on the limits for experiment on mice. 10 min exposure was the time that appeared best suited for the treatment of a tumor requiring an externalization from the animal maintained under general anesthesia. The delay between treatments (10 days) was the time required for animal recovering and proper healing. This protocol has previously shown perfect tolerance by healthy pancreatic tissue. During all treatment procedures, anesthetized mice were placed on a temperature regulated silver plate, pancreas was externalized thanks to a surgical procedure and at that time, caliper measurements were done. Either the helium flow alone or the plasma was delivered through the capillary tip positioned at a distance of two millimeters above the tumor during 10 minutes (**Figure 1a**).

In the last group, mice received a NTP/GEM combined treatment. Mice received gemcitabine 24 hr before NTP exposure because this administration schedule was previously shown to be the most efficient in a context of radiotherapy treatment [16] and considering that NTP treatment potentially induces chain processes that might be similar with radiotherapy [17] as mentioned earlier. The therapeutic schedule was the same for gemcitabine alone and NTP alone groups (i.e. every 5 days for gemcitabine, every 10 days for NTP) (**Figure 1b**).

At day 36 post induction, mice were euthanized and the tumor size measured with a caliper, volume (V in mm^3^) being calculated using the formula for an ellipsoid V = 4/3pi(a/2*b/2*c/2). a, b, c being the full length of the three axes.

### Bioluminescence Imaging

BLI allows real-time and non-invasive imaging of tumor evolution. BLI is based on detection of photon released through chemical reactions catalyzed by luciferase and depending on ATP and O_2_. As a consequence, BLI intensity is closely related to tumor activity and size at the time the tumors are in aerobic conditions. During tumor growth, metabolism shifts towards hypoxia and the bioluminescence as a biomarker becomes no more relevant.

BLI mice imaging was performed before the first treatment (D3) and then weekly during treatment using the IVIS-LuminaII imaging system (Caliper Life Sciences, Roissy, France). Each mouse was intraperitoneally injected with luciferin potassium salt (Promega, France) at a dose of 100 mg/kg and imaged after 5 minutes. Animals under gaseous anesthesia were placed on a temperature controlled warm bed (37°C) inside the dark box of a high sensitivity *Charge* Coupled Device camera cooled to −90°C. Acquisition settings (binning and duration) were set up depending upon tumor activity. Region of interest was drawn manually around the tumor area, and the light (photons/sec) emitted from the region of interest was measured using Living Image software (Caliper Life Sciences, Roissy, France).

### Cell Growth Assay

The cytotoxicity of NTP and Gemcitabine was investigated using MTT assay and BLI.

Briefly, cell viability was determined by measuring 3-(4,5-dimethylthiazol-2-yl)-2,5-diphenyltetrazolium bromide (MTT) dye absorbance by living cells. Cells were seeded in 96 well culture plates and incubated overnight before being treated with NTP or Gemcitabine. After a 48 hr incubation period, a MTT solution (2.5 mg/ml in PBS) was added to the culture medium, and cells were incubated for 4 h. Formazan crystals resulting from MTT reduction by viable cells were dissolved by the addition of an equal volume of solubilizing solution (10% SDS in DMSO/acetic acid 99/1) in each well. The absorbance of formazan was measured at a wavelength of 570 nm.

For *in vitro* BLI imaging, cells were seeded in 24 well culture plates and incubated overnight before being treated with NTP or gemcitabine. After a 48 hr incubation period, 300 µg/ml luciferin potassium salt (Promega, France) were added in each well and the BLI was measured after 5 min incubation at 37°C using IVIS-lumina II imaging system (Caliper Life Sciences, Roissy, France). BLI intensity expressed in photons/sec in each well was normalized to the non-treated cell level.

### Necropsy and Pimonidazole Assay

Pimonidazole as a 2-nitroimidazole compound is reductively activated at low-oxygen concentrations. Pimonidazole adducts bind to cell molecules that possess free thiol groups then accumulate *in vivo* and indicates tissue hypoxia directly at the cellular level. Hypoxyprobe® (pimonidazole) was obtained from the Natural Pharmacia International, Inc. (Research Triangle Park, NC) and was dissolved in 0,9% saline solution at a concentration of 120 mg/ml. Each animal received 120 mg/kg Hypoxyprobe® i.p. one hour prior to be sacrificed. Hypoxic accumulation of pimonidazole was determined by immunohistochemistry.

### Immunohistochemistry

Tumors were embedded in Tissue Tek and frozen sections fixed with 4% paraformaldehyde in Phosphate Buffered Saline (PBS) for 15 min at room temperature. After being washed with PBS, slices were incubated in 5% Foetal Bovin Serum (Invitrogen) and 0,1% Triton-X (Sigma-Aldrich) to inhibit nonspecific binding of antibodies. Primary antibodies were applied, and sections were incubated overnight at 4°C. The primary antibodies used were anti-luciferase rabbit polyclonal (1∶100, Abcam, Cambridge, UK) and anti-pimonidazole mouse monoclonal (1∶100, Hypoxyprobe, HPI, Inc., USA). Slices were then washed with PBS, Fluorescein IsoThioCyanate (FITC) conjugated anti-mouse IgG and Tetramethyl Rhodamine Isothiocyanate (TRITC) conjugated anti-rabbit IgG (1∶500) were added and the tissue sections were incubated for 60 min at room temperature. Slices were coverslipped with DAPI mounting medium and visualized on a ZEISS LSM 510 Meta confocal microscope equipped with Axiovision software (CARL, ZEISS SAS, Le Pecq, France).

### Data Analysis

Results are expressed as means ±SEM. Statistical significance was determined by Mann-Whitney test. LD 50 was determined according to the Hill slope method (GraphPad Prism 5.0, La Jolla, CA). Differences were considered significant at *p* values <0,05.

## Results

### NTP and Gemcitabine Present a Significant Antitumor Effect *in vitro*

Lentiviral particles were used to transfer the firefly luciferase and EGFP genes to MIA PaCa2 cells. Two weeks after viral transduction, flow cytometry analysis showed that 100% of virus-treated MIA PaCa2 cells expressed EGFP (data not shown), indicating homogenous modification of cells. *In vitro* analysis of the luciferase activity revealed a 250 fold increase for virus-treated MIA PaCa2 cells (MIA PaCa2-luc cells) compared to parental cells (data not shown).

**Figure 2:**
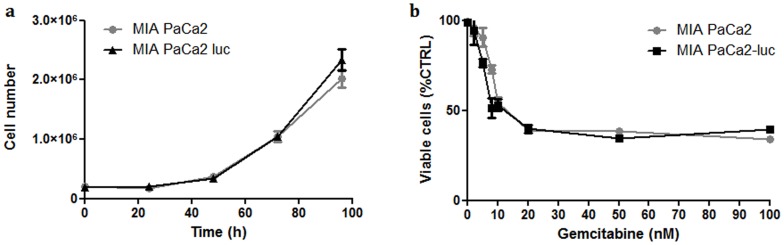
Impact of the transduction on MIA PaCa2 cells; a) *In vitro* cell growth of transduced and non transduced MIA PaCa2 cells. Each point represents the mean (±SEM), *n* = 3 for each group. b) Cells response to gemcitabine alone *in vitro*. Cell viability was determined 24 h after treatment by MTT and was normalized to untreated cells. Each point represents the mean (±SEM), *n* = 5 for each group.

**Figure 3:**
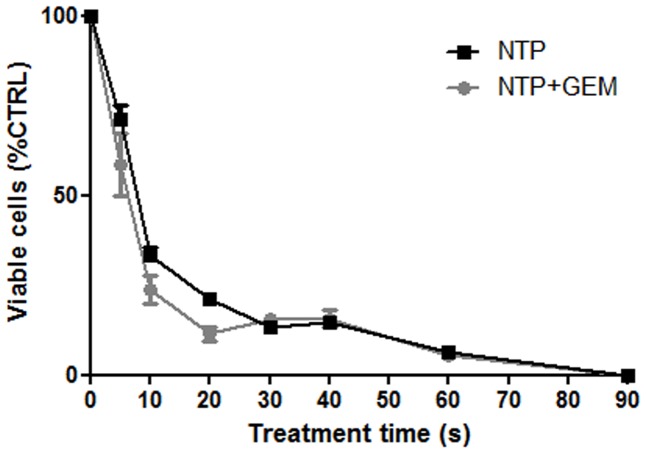
Inhibitory effect of NTP on cell proliferation *in vitro* in MIA PaCa2-luc cells. Cells were treated with NTP alone or in combination with gemcitabine. Cell viability was determined 24 h after treatment by bioluminescence and was normalized to untreated cells. Each point represents the mean (±SEM), *n* = 5 for each group.

The impact of the transduction with firefly luciferase expressing viral particles on cell growth *in vitro* was assessed during 96 hours and revealed no difference between both cell lines (MIA PaCa2 and MIA PaCa2-luc cells), figure 2a. Moreover, evaluation of treatment responses on non transduced and transduced cells was realized using gemcitabine. The cell viability and metabolism assays showed no modification of the two cells lines responses (Figure 2b). Using BLI, both treatments (NTP and gemcitabine) present a significant antiproliferative effect on pancreatic cells and the IC_50,_ as determined using Hill slope method, was 9 s and 10 nM for NTP and gemcitabine respectively (Figure 3). Combined treatment NTP/Gemcitabine (5 nM) lead to an IC_50_ of 4 s. Considering the interesting antiproliferative potential of NTP and gemcitabine on MIA PaCa2-luc cells, the antitumor evaluation of these treatments was done *in vivo* on orthotopic xenografts.

### Bioluminescence Limitations are Linked to the Hypoxic Characteristics of MIA PaCa2 Luc Tumors

**Figure 4:**
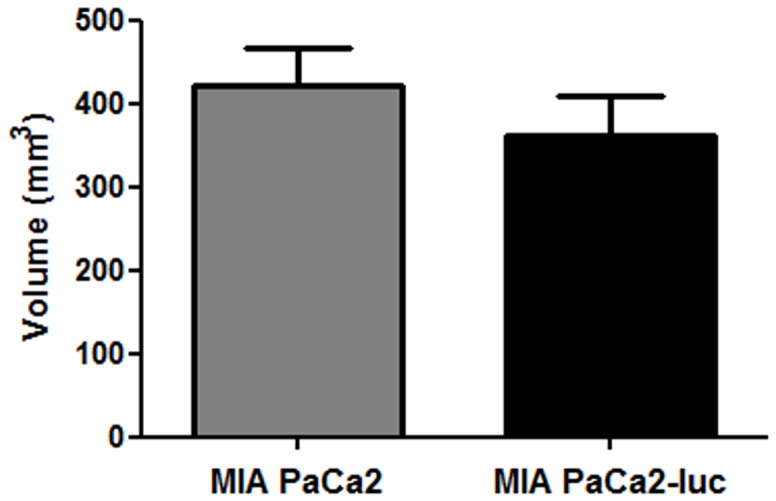
Tumor volume after orthotopic injection of transduced or non transduced MIA PaCa2 cells. Tumor volumes were measured with a caliper at the day of euthanasia (D36). The columns represent the means (±SEM), *n* = 7 for each group.

**Figure 5:**
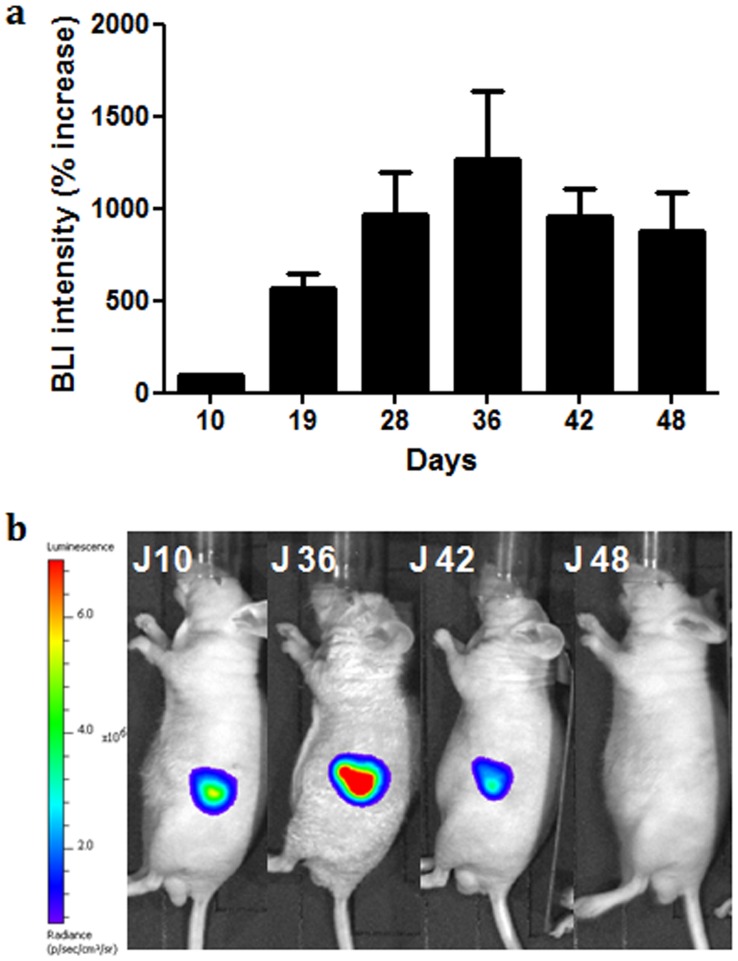
Growth of MIA PaCa-2 tumors xenografted into nude mice. a) Tumor volumes monitored by bioluminescence for the 48 days of the experiment. Values represent means ±SEM, *n* = 9 for each group. b) Exemplary results from *in vivo* BLI, showing one non treated mouse with loss of BLI signal.

Before assessing the antitumor effect of gemcitabine and NTP treatments, we needed to characterize the orthotopic tumor model induced with MIA PaCa2-luc cells. Considering weight and tumor volume, no significant difference in tumor growth was observed at D36 between the groups of nude mice that received parental or transduced cells (figure 4). Next, transduced cells were used for assessment of tumors BLI intensity from D10 to D48 (figure 5). An increase of BLI intensity was observed from D10 to D36 reflecting tumor growth, but a decrease of BLI intensity was observed from D42. This evolution of light emission which is not in accordance with the progression of tumor volume could be associated to several mechanisms including either a loss of luciferase gene expression or hypoxia, since BLI is an imaging modality strictly dependent upon cells metabolism.

**Figure 6:**
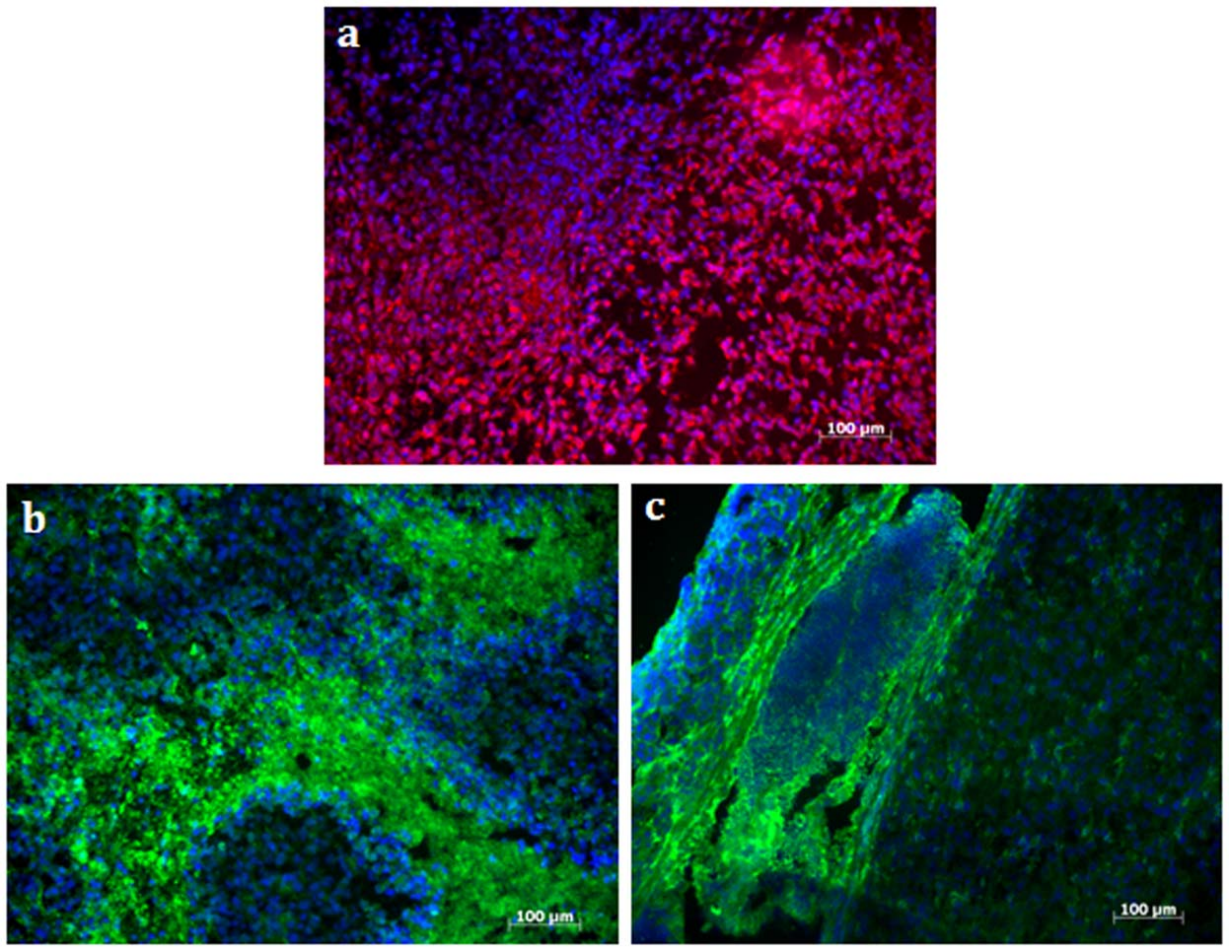
Immunohistochemistry of pancreatic tumor sections 48 days post MIA PaCa2-luc cell injection (Bar = 100 **µm).** a) Anti-luciferase antibody labeled with TRITC (red) confirmed luciferase expression in tumor sections; b and c) *In situ* detection of hypoxic tumor regions by using anti-pimonidazole antibody labeled with FITC (green). b) An intensive hypoxic tumor regions can be seen at the center. Positive immunosignals were also seen at the periphery c).

To visualized the persistence of luciferase protein in the tumor cells, mice were euthanized on D48 and tumor excised to perform immunohistochemistry with an anti-luciferase antibody. As displayed in figure 6a, all cells presented a positive labeling of luciferase on the whole tumor section. To localize hypoxic cells in the tumors, a pimonidazole adducts staining was performed leading to a positive immunosignal in the center and the periphery of the tumor (figure 6b). From these observations, the assessment of primary tumor growth with quantitative BLI was considered as not relevant beyond 36 days after tumor induction.

### Gemcitabine Reduces Tumor Proliferation

**Figure 7:**
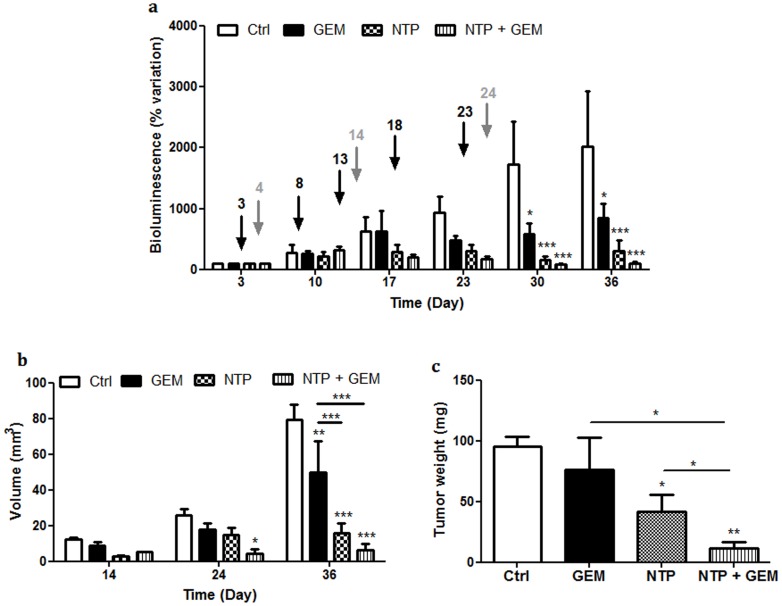
Plasma gun and gemcitabine treatments effects on tumor growth in an orthotopic pancreatic model. Three days after tumor induction mice were randomly assigned into 4 groups: control (CTRL), gemcitabine (GEM), plasma gun (NTP) and bitherapy (NTP+GEM), eight mice per group. a) Tumor evolution followed by bioluminescence. The gray arrows represent NTP treatments and the black arrows gemcitabine treatments days. Tumor BLI was normalized to signal at D3; b) tumor volume was determined using a caliper at two times of the plasma treatment and on day of euthanasia; c) tumor weight at the end of the study. Each point represents the mean (±SEM). *p<0,05, **p<0,01, ***p<0,001.

The gemcitabine antitumor efficacy was tested *in vivo* on orthotopic pancreatic tumors induced with MIA PaCa2-luc cells. BLI results are presented in **figure 7a**. In the group treated with saline and gas flow (CTRL group), an increase of BLI was observed along the weeks while in the group treated with gemcitabine (GEM group) a stabilization of BLI was obtained from D17. The observed antitumor effect was confirmed by caliper measurements at the end of the study (D36). Volumes of non-treated tumors were 80 mm^3^ as compared to 50 mm^3^ for treated tumors (**figure 7b**). Tumor weights lead to similar results with 96 mg and 76 mg respectively (**figure 7c**). At D36, the volume difference between gemcitabine-treated and non-treated tumors was significant (p<0,01) whereas the weight difference was not significant.

### NTP Treatment Produces a Greater Effect than Gemcitabine on Tumor Growth

NTP and gemcitabine antitumor effects were then compared on MIA PaCa2-luc cell-induced pancreatic tumors. Results obtained from BLI imaging performed once a week are presented in **figure 7a**. In the CTRL group, an increase of BLI was observed, while in the group treated with NTP (NTP group) a highly significant stabilization was induced by the treatment (p<0,001 at D30). The observed antitumor effect was confirmed from caliper measurements. At D36, volumes of non-treated tumors were 80 mm^3^ as compared to 16 mm^3^ for treated tumors (**figure 7b**). Tumor weights showed similar variations with 96 mg and 42 mg respectively (**figure 7c**). So NTP was shown to be more effective than gemcitabine 200 mg/kg.

### The Combination Gemcitabine/NTP Enhances the Antitumor Effect

By inhibiting the DNA synthesis, gemcitabine has been shown to enhance the cytotoxic activity of radiation [11]. Our next goal was then to test the combined effects of NTP and gemcitabine treatments to enhance the antitumor effect without increasing toxicity. When combining NTP and GEM, BLI intensity was significantly lower than CTRL group (**Figure 7a**). At D36, a significant difference of tumor volumes was observed for the group treated with the bitherapy (NTP+GEM group) (6 mm^3^) as compared to CTRL group (80 mm^3^). Similar results were obtained for tumor weights with 12 mg for NTP+GEM group and 96 mg for CTRL group (**Figure 7c**).

When comparing NTP+GEM and NTP groups, a significant difference of tumor weights, 12 mg and 42 mg respectively, was observed at D36 (p = 0,03). This observation shows that the plasma gun antitumor effects are increased by 33% when it is associated with gemcitabine (**figure 7c**).

## Discussion

This experimental work reports that plasma gun, alone and more especially in combination with gemcitabine, induces a significant reduction of tumor growth in a mouse orthotopic model of human pancreatic cancer.

Tumor of the exocrine pancreas mainly ductal adenocarcinoma, the most common form of pancreatic cancer [7], is rarely curable and has an overall survival rate of less than 5%. Moreover, chemotherapy and radiotherapy are ineffective in most cases, so new therapeutic approaches and *in vivo* models offering high predictivity for translational research are necessary. In this context, we evaluated a new antitumor strategy based on non-thermal plasma which allows a local treatment. Indeed, NTP can be applied at the end of a small catheter and has demonstrated significant antitumor activity on various cell lines *in vitro* including colorectal and melanoma cells [3], [18]–[20]. This antitumor activity is related to a major cell death induction resulting from high rate generation of ROS in the vicinity of the cells [3], [21]. With the Plasma Gun, the ROS are mainly produced at the capillary outlet where energy transfer occurs between the helium plasma and the ambient air.

This local treatment allows to avoid systemic side effects because only adjacent tissue of the tumor are exposed. Moreover, some studies showed a major sensitivity of tumor cells as compared to healthy tissues [6], [19]. This targeted effect could be explained by a better ROS tolerance of normal cells than tumor cells which have a higher basal level of ROS [22].

In our study, we investigated initially *in vitro* effects of gemcitabine and/or NTP on a representative cell line of ductal adenocarcinoma. An activity of gemcitabine in agreement with previous results [23]–[25] and a significant effect of NTP were observed. Given these encouraging results *in vitro*, and considering the good tolerance of plasma [18], we further investigated the potential antitumor properties of NTP treatment *in vivo*. Trying to provide more pertinent preclinical data, we have developed our approach in a context of translational research using a relevant tumor model and a clinically based treatment strategy. The first requirement was the development of an orthotopic pancreatic tumor model in mouse with mainly metastatic potential [26], [27] and hypoxic component [28], thus closely mimicking the pathophysiology of human pancreatic cancer [29]. Despite the different morphology of human and mouse pancreas similar patterns of metastasis were observed in agreement with published data [29]. Indeed, at late times (D45) 70% mice exhibited spleen, peritoneum metastasis and few foci in liver, kidney, stomach, intestines and diaphragm (data not shown), supporting a preservation of cells properties following transduction. To date, a powerful and widely used imaging modality in oncopharmacology for orthotopic tumors is BLI, an imaging based on a gene expression dependent upon metabolism (O_2_ and ATP). In aerobic conditions, bioluminescence intensity is closely dependent upon cell number and correlates with cell proliferation [30], [31]. In this study, our data showed a decreased bioluminescence beyond D36 in the control group, leading to two hypotheses: either loss of luciferase gene expression or hypoxic areas in the tumor. An anti-luciferase immunolabelling confirmed the presence of luciferase protein in the whole tumor. However, antipimonidazole immunolabelling revealed hypoxia both at the center and at the periphery of the tumor. This suggests that hypoxia is involved in the observed reduction of the bioluminescent signal. This hypoxia pattern was already reported in a preclinical model of pancreatic tumor [32] as well as in human pancreatic cancers which are characterized by a low oxygen tension [32]. The decrease of BLI along the tumor growth associated to the progression of hypoxia is an important limitation to consider for a relevant therapeutic drug evaluation that requires quantitative imaging. So, use of BLI for a longitudinal study of the primary tumor must be considered only during the time it is proportional to tumor size (<36 days for the present study). If it is necessary to document later stages for the primary tumor, other imaging modalities such as ^18^F-FDG Positron Emission Tomography with satisfactory sensitivity [33], but a moderate specificity [34] is an alternative to get quantitative data since scintigraphy with ^111^In-pentetreotide or other biomarkers such as Vasoactive Intestinal Peptide [35] and bombesine [36] appear not to be efficient quantitative modalities in mice models. Another strategy could be the use of 3D infrared fluorescence imaging of integrins (αvβ3 and αvβ5) that are overexpressed by both tumors and neo-endothelium [37]. However quantitation in 3D infrared fluorescence still requires some technological improvements. So, considering that our study can be achieved within the 36 days period and since it was possible to perform tumor size measurement at each treatment times, bioluminescence was considered as a suited modality to provide quantitative data. At the end of the study (D36), bioluminescence values were compared with tumor weights and volumes to confirm that at this time bioluminescence can be still considered as a relevant biomarker of tumor proliferation.

When NTP is applied directly on the tumor, a significant antitumor activity is obtained. Previous studies have showed such an *in vivo* antitumor effect of plasma on subcutaneous tumors [1], [4], [5] only. This paper reports for the first time a high antitumor activity of fibered plasma on an orthotopic model. We have previously reported that plasma antitumor activity was linked to a high rate generation of ROS in the vicinity of the cells and an apoptosis induction [3]. To improve NTP antitumor activity, recent *in vitro* studies suggest the potential interest of the combination of NTP with chemotherapy. Indeed, a beneficial effect of the combination of cyclophosphamide with NTP has been reported even if mechanisms involved are still not elucidated [6]. We chose to associate NTP treatment to gemcitabine, a classically used chemotherapy for pancreatic tumor.

This association could be of valuable interest because in addition to its cytotoxic effect, this nucleoside analogue is a potent radiosensitizer of rodent and human tumor cells, including pancreatic tumors [38]–[40]. It has been hypothesized that gemcitabine could induce Deoxyadenosine Triphosphate (dATP) depletion and cause an accumulation of MIA PaCa-2 cells in early S phase [41] as a result of the inhibition of DNA synthesis, which may play a role in enhancing radiosensitivity [11]. These mechanisms associated to the effect of NTP on cell cycle, especially the multiphase cell cycle arrest observed after treatment [3], [42], [43], could increase the antitumor activity *via* an activation of the apoptotic pathway, indeed the generation of ROS in the vicinity of the cell induced various type of DNA damages which lead to the cell cycle arrest and finally to an apoptosis induction. We report here that gemcitabine administered 24 h before NTP allows to reduce the tumor proliferation. This administration schedule was previously shown to be the most efficient in a context of radiotherapy [16]. Under the conditions used for our experiments, and in comparison with NTP group, a 33% decrease in tumor weight was obtained by combined therapy. As previously observed in radiotherapy, this improvement of antitumor activity using Gemcitabine/NTP combination may be the result of a modulation of cell cycle facilitating difluoro-dCTP incorporation in DNA [44].

In conclusion, our results suggest that fibered NTP could be a new therapeutic strategy against tumors, more especially when used in combination with antitumor drugs. The excellent tolerability and the effectiveness of NTP towards MIA PaCa2-luc cells *in vivo*, together with the ability to deliver NTP via a small capillary open new interesting perspectives for loco-regional or *in situ* applications. For example, treatment of intraductal papillary mucinous, tumors of the pancreas, colorectal and bronchopulmonary recurrences. Development of the plasma source and optimization of the administration to enhance the effects of the Gemcitabine/NTP combination is our next goal together with the characterization of the biological processes (i.e. apoptosis; necrosis, necroptosis and angiogenesis) induced by plasma treatment. Moreover translation from orthotopic model in mice to dogs with pancreatic tumors will be an important step to assess the antitumor potential of plasma in relevant models. In this spontaneous tumor model, fibered plasma will be associated to interventional imaging using a near infra red fluorescence probe to detect tumor foci.
